# Bortezomib in Kidney Transplantation

**DOI:** 10.1155/2010/698594

**Published:** 2010-09-27

**Authors:** Rajeev Raghavan, Abdallah Jeroudi, Katafan Achkar, A. Osama Gaber, Samir J. Patel, Abdul Abdellatif

**Affiliations:** ^1^Department of Medicine, Baylor College of Medicine, Houston, TX 77030, USA; ^2^Division of Nephrology, Baylor College of Medicine, Houston, TX 77030, USA; ^3^Department of Medicine, The Kidney Institute and The Methodist Hospital, Houston, TX 77030, USA; ^4^Division of Nephrology, The Kidney Institute and The Methodist Hospital, Houston, TX 77030, USA; ^5^Department of Surgery, The Methodist Hospital, Weill Cornell University, Houston, TX 77030, USA; ^6^Department of Pharmacy, The Methodist Hospital, Weill Cornell University, Houston, TX 77030, USA

## Abstract

Although current therapies for pretransplant desensitization and treatment of antibody-mediated rejection (AMR) have had some success, they do not specifically deplete plasma cells that produce antihuman leukocyte antigen (HLA) antibodies. Bortezomib, a proteasome inhibitor approved for the treatment of multiple myeloma (a plasma cell neoplasm), induces plasma cell apoptosis. In this paper we review the current body of literature regarding the use of this biological agent in the field of transplantation. Although limited experience with bortezomib may seem to show promise in the realm of transplant recipients desensitization and treatment of AMR, there is also experience that may suggest otherwise. Bortezomib's role in desensitization protocols and treatment of AMR will be defined better as more clinical data and trials become available.

## 1. Introduction

Kidney transplantation is the treatment of choice for most patients with stage five chronic kidney disease (CKD). The risk of death is less than half of that for dialysis patients regardless of the immunosuppression protocol used [1]. Furthermore, most recipients acknowledge improved quality of life. It is not surprising that the demand for donor kidneys continually outpaces the supply. The United Network for Organ Sharing (UNOS) has over 80,000 patients on the kidney transplant waiting list, many of whom are highly sensitized. Data obtained from the UNOS (2001–2008) showed that the rates of transplantation for living donor (LD) and deceased donor (DD) by panel reactive antibody (PRA) status are less than 16% per year for patients with PRAs of 10% to 80%, and less than 8% for patients with PRAs more than 80%. Thus, sensitized patients with any level of PRA are difficult to transplant and have longer waiting times on the transplant list [2]. Strategies for removing or decreasing preformed antibodies in these patients are termed desensitization. Literature review demonstrates 1-year allograft survival between 69% and 96% for desensitizieted patients [3]. 

 The rejection risk for all patients in the first year post transplant is less than 12% based on the 2009 USRDS database [4]. Highly sensitized transplant recipients, regardless of the desensitization protocol used, are at increased risk for AMR. Both desensitization and AMR are managed with the similar therapeutic arsenal; however protocols are center-specific and there are no consensus guidelines [5]. The two desensitization protocols for which clinical efficacy has been demonstrated are high-dose IVIG or low-dose IVIG with either plasmapheresis (PP) or immunoadsorption [6, 7]. Additionally, some transplant centers may add intravenous steroids, rabbit antithymocyte globulin (rATG), or rituximab [8]. As mentioned above, these modalities are variably effective in decreasing reactive antibody levels [9–11]. 

**Figure 1:**
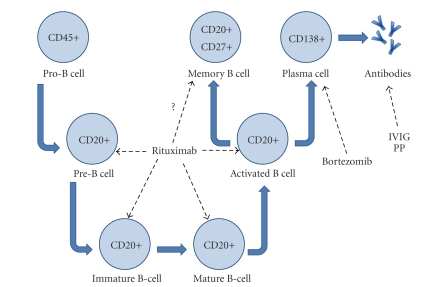
A simplified, conceptual diagram of the targets of current therapeutic modalities for pre-transplant desensitization and treatment of antibody mediated rejection. The dashed arrows indicate the sites of action for the therapeutics. Rituximab exerts its effects on CD20+ B-cell lines with absence of activity against pro-B cells and plasma cells and questionable activity against memory B cells. Bortezomib targets plasma cells which elaborate the antibodies implicated in donor-specific antibodies and antibody-mediated rejection while the antibodies produced are targeted with intravenous immunoglobulin (IVIG) and plasmapheresis (PP).

There is concern that the role of plasma cells in mediating humoral rejection is not adequately addressed [9]. Since plasma cells do not express CD20, they are not depleted by rituximab's ability to deplete CD20 positive B-cell line members as detailed in (Figure 1). There is one variant of AMR in which over 30% of infiltrating cells are mature plasma cells, and once diagnosed graft survival is generally less than one year post diagnosis [12]. Hence, it is of importance to target this cell lineage in desensitization and AMR treatment strategies. 

Reservations were expressed in the literature that plasma cells were unaffected by current desensitization protocols. The study by Ramos et al. confirmed these ruminations. The group conducted a study where the spleens of patients receiving desensitization were histologically compared to control spleens for their levels of different B-cell line members [13]. The study showed that levels of naïve B cells (CD20+ and CD79+), memory B cells (CD27+), and plasma cells (CD138+) in the spleens of patients desensitized with PP and low-dose IVIG did not differ significantly from control spleens. It was also noted that despite the addition of rituximab to the PP and IVIG protocol, the amount of memory B cells and plasma cells were still comparable to controls. Combination therapy in the study (PP, low-dose IVIG, rituximab, and rATG) did show a small reduction of memory B cells, but plasma cell levels were still on par with controls. This study confirmed the reservations expressed in the literature that plasma cells were unaffected by current desensitization protocols [9, 13]. 

Bortezomib (Velcade, Millennium Pharmaceuticals, Cambridge, MA) depletes plasma cells via proteasome inhibition [8]. In 2008, investigators at the University of Cincinnati published their experience of six patients with AMR and donor-specific antibodies (DSA) elevation post transplantation who had reversal of AMR with a single cycle of bortezomib [14]. Several other transplant centers have since utilized bortezomib for treatment of AMR with varying success [14–20]. Herein, we review the current body of literature regarding using bortezomib in pretransplant desensitization and treatment of AMR.

## 2. Bortezomib Biological Effect

Bortezomib was first synthesized in 1995. After just seven years, the Food and Drug Administration (FDA) approved this drug as treatment for multiple myeloma, a plasma cell dyscrasia, which remains its only approved indication. A phase 3 multicenter trial published in the New England Journal of Medicine in 2008 demonstrated marked improvement in outcomes for newly diagnosed myeloma patients who received bortezomib in addition to the treatment standard of melphalan and prednisone. This 682-patient randomized trial demonstrated the superiority, along with safety and efficacy of Bortezomib. Now this drug is first-line treatment in patients with newly diagnosed myeloma who cannot receive immediate autologous stem cell transplantation [21]. The drug has been used off label in the transplant setting since 2005 both to reduce DSAs in highly sensitized patients and as an adjunct therapy for AMR. 

Bortezomib (C_19_H_25_BN_4_O_4_) has a central boron atom which binds the catalytic site of the 26S proteasome with high affinity and specificity. Present in all cells, the proteasome degrades ubiquitinylated, abnormal, and misfolded proteins; thus regulating protein expression and function [22]. Simply put, proteasome inhibition during mitosis inhibits the degradation of cell-cycle regulatory proteins resulting in cell-cycle death via apoptosis. One such regulatory protein is NFkB, which has an important role in controlling cell cycle progression, loading of Class I MHC molecules, cell adhesion, and activation of cytokines [23]. This molecule is inhibited by transcription factor IkB. Bortezomib's interference with these two molecules leads to the accumulation and aggregation of unfolded proteins and eventual plasma cell apoptosis.

Both in vitro and in vivo (murine and human) studies have noted that this drug has a propensity to cause apoptosis of CD138+ plasma cells [16, 24]. Bortezomib also exerts numerous indirect effects on circulating B cells and T_H_ cells; for example, it may lead to blockade of T-cell cycling leading to apoptosis of T_H_ cells and reduction of bone marrow interleukin-6 can decrease B-cells numbers [21].

## 3. Bortezomib Pharmacokinetics, Pharmacodynamics, and Side Effects

The pharmacokinetics of bortezomib can be characterized by rapid and wide distribution, a prolonged elimination half life, and hepatic cytochrome P-450 (CYP) isoenzyme metabolism [25]. After a rapid distribution half life of approximately 10 minutes, peak plasma bortezomib concentrations range 60 to 120 ng/mL following repeated doses of 1 to 1.3 mg/m^2^. Total body clearance decreases from 102 to 112 L/h after the first dose and from 15 to 32 L/h following repeated doses [26]. Subsequent elimination half-life ranges from 40 to 190 hours. A high volume of distribution (500 to 1800 L/m^2^) in patients with multiple myeloma is suggestive of extensive distribution to peripheral tissues. In vitro studies indicate bortezomib metabolism to occur primarily via hepatic oxidation by CYP3A4, CYP2C19, CPY1A2, and to a lesser extent CYP2D6 and CYP2C9, to inactive metabolites. While inhibitors and inducers of these isoenzymes are commonly seen especially in organ transplant populations, clinically significant interactions with bortezomib and enzyme inducers have not been reported. Concomitant administration of ketoconazole has been shown to increase systemic bortezomib exposure by 35% with corresponding increased proteasome inhibitory activity, although side effects were similar to those not receiving ketoconazole [27]. On the other hand, ascorbic acid (Vitamin C) has been shown to interfere with bortezomib's proteasome inhibitory activity through alternative mechanisms [28]. The pharmacodynamic profile of bortezomib provided a basis for its current recommended dosing regimen. Following intravenous administration, maximum percent inhibition of 20S proteasome is observed after 5 minutes, reaching a mean of 70–84% inhibition [26]. With decreases in drug concentration, 20S proteasome inhibition is reversed with mean inhibition declining to 22–48% at 48 hours and returning to baseline at 72 hours post administration. Pharmacodynamic properties were similar between 1.0 and 1.3 mg/m^2^ dose regimens [26]. Adverse effects are reported in Phase II and Phase III studies from multiple myeloma and mantle cell lymphoma populations. The main adverse effect of this drug is neurotoxicity which manifests as a dose-related peripheral sensory neuropathy that may occur in about 30% of treated patients. This neuropathy can often be severe but is reversible with discontinuation of the drug. Severe events (National Cancer Institute Common Toxicity Criteria grade 3 event) also noted with bortezomib therapy include thrombocytopenia (28%) and neutropenia (11%) which are usually managed with standard approaches. Thrombocytopenia due to bortezomib has been observed to occur in patients primarily with low baseline platelet levels and resolves upon drug discontinuation. Other commonly reported side effects include nausea (55%), diarrhea (44%), and fatigue (12%); the gastrointestinal disturbances are usually mild and managed easily with standard approaches [29]. In the largest series of kidney transplant recipients to date, Walsh et al. report a similar pattern of adverse effects, including low-grade gastrointestinal side effects, mild to moderate anemia, neutropenia, and thrombocytopenia, and primarily mild cases of peripheral neuropathy in patients undergoing treatment for desensitization and humoral rejection [30]. 

Dosing of bortezomib is similar regardless of the route of application, it does not require renal or hepatic dosing adjustments, and the drug is no longer detectable within 30 minutes of injection [22]. 

## 4. Bortezomib Use in Kidney Transplantation

To our knowledge, this drug has never consistently been used in nonkidney transplant protocols. There are several published case reports and case series detailing bortezomib's application in kidney transplantation. Many centers now use velcade-based protocols for highly sensitized patients.

### 4.1. Bortezomib in Desensitization Protocols

Table 1 lists the published data of this drug's use in kidney transplantation. The first published data is from University of Cincinnati by Idica et al. [17] in 2008. Thirteen highly sensitized patients received this drug and all had reduction in the normalized mean fluorescent units (MFI) of the donor-specific antibodies; ten of whom (77%) had significant decrease in DSA. 

Trivedi et al. reported on 11 patients with a posttransplant anti-HLA antibody titer greater than 1000 MFI, but without acute rejection. The use of bortezomib with plasmapheresis was successful in decreasing antibody levels to under 1000 MFI within a median time of 24 days from treatment initiation in all but two patients [15]. Both of these two patients had a peak MFI greater than 10,000. Overall the study suggests that bortezomib can be used to decrease DSA levels with minimal toxicity [15]. Four of 11 patients had reappearance of anti-HLA antibodies despite initial effective reduction with one cycle of bortezomib. The authors suggested that certain patients may need more than one cycle of treatment to decrease DSA levels. With clinically stable patients, the study's findings neither argues for nor against bortezomib's ability to affect the clinical course of graft rejection, but the study does point to the possible role that bortezomib can play in decreasing DSA levels that are implicated in AMR. 

In the case series by Wahrmann et al., the group used two cycles of bortezomib for pre-transplant desensitization for two highly sensitized kidney recipients [18]. The first cycle of bortezomib was given alone followed by a cycle of bortezomib with dexamethasone as dexamethasone has been shown to enhance treatment efficacy in multiple myeloma patients. In the two patients, PRA decreased from 87% to 80% in patient 1 and 37% to 13% in patient 2. Despite the mild decrease in PRA levels, bortezomib therapy led to more than 50% decrease in the levels of anti-HLA antibodies triggering C4d deposition on single antigen Luminex beads as measured in MFI after 6 months of followup. This suggests that bortezomib may have a role in decreasing complement fixation especially as C4d is one of the histological markers leading to the diagnosis of AMR [18]. Yet, bortezomib's mild effect on PRA for pre-transplant desensitization may suggest the need for adjunct modalities that target antibodies such as PP and IVIG as well as further exploration of bortezomib in transplant desensitization. 

### 4.2. Bortezomib in Rejection Protocols

In the first study to use bortezomib as an antirejection modality, 6 kidney transplant recipients with AMR and acute cellular rejection (ACR) refractory to plasmapheresis, IVIG, and/or rATG, and/or rituximab were treated with bortezomib [14]. Bortezomib therapy led to prompt rejection reversal (within days to weeks) and in all the cases, there was improved renal function and reduction in DSA levels. Recurrent rejection episodes in 2 patients were suppressed for up to 5 months; furthermore, the anti-HLA antibody with the highest levels (immunodominant DSA) were decreased by more than 50% within 14 days and remained suppressed for up to 5 months [14]. As the first positive study of incorporating bortezomib as a suppressor of DSA in the treatment of AMR, others groups have emulated this strategy. 

Perry et al. analyzed the effects of rATG, IVIG, rituximab, and bortezomib on enriched populations of bone marrow derived plasma cells and indicated that only bortezomib was successful in causing plasma cell apoptosis and completely blocking anti-HLA IgG secretion against all specificities in vitro compared to controls [16]. In comparing the bone marrow of 2 positive cross-match kidney recipients at the time of AMR and at 1 week after treatment with bortezomib for AMR, in vivo data showed a decrease in the percentage of bone marrow plasma cells, a decrease in antibody production (including DSA), and a decrease in the number of plasma cell allospecificities in the bone marrow aspirates obtained after bortezomib treatment. With resolution of AMR and normal kidney function at one year post transplant, the study suggests that bortezomib can target plasma cells implicated in AMR [16].

In the study by Walsh et al., two patients undergoing acute AMR with high DSA and positive Cd4 staining on biopsy two weeks after kidney transplantation were treated with a multiday regimen consisting of PP paired with methylprednisolone and bortezomib along with a single dose of rituximab [19]. The theoretical rationale behind this protocol was that bortezomib would target plasma cells, rituximab would target plasma cell production from the pool of memory B-cells, and plasmapheresis would remove antibody levels creating a demand for increased antibody production to potentiate the effect of bortezomib's proteasome inhibition. In addition, plasmapheresis was used to remove pre-existing antibodies that may have been circulating for weeks and thus measure antibody levels that would correlate with the magnitude of the antibody producing plasma cell population. By nearly 14 days after treatment, DSA levels had dropped significantly as well as repeat biopsy showed faint peritubular capillary C4d labeling and decreased glomerular C4d deposition. For patient 1, DSA remained below detectable thresholds for 6 months following bortezomib treatment. For patient 2, DSA remained below detectable thresholds for 2 months before a rebound in DSA titers were observed. A repeat cycle of PP, rituximab, and bortezomib treatment was given showing undetectable DSA within the first week of retreatment [19]. The group concluded that bortezomib-based regimens may be beneficial in rapid DSA elimination in the setting of acute AMR as an adjunct to commonly used modalities such as PP.

In the study by Sberro-Soussan et al., the group came to an opposite conclusion of bortezomib efficacy in four patients at least 1 year out from renal transplantation [20]. In these four patients experiencing subacute AMR, no significant decrease in DSA intensity as measured by MFI occurred despite use of PP, IVIG, rituximab, and bortezomib in 150 days of followup. In addition, bortezomib's activity on long-lived plasma cells as measured by observing any decrease in antiviral antibodies (antihepatitis B surface antigen) and total IgG was not demonstrated as neither decreased significantly with a cycle of bortezomib. The group postulated that lack of activity against DSA may have been secondary to a long period of DSA stability following transplant as bortezomib was administered nearly 1 year or more after transplantation of these patients. They concluded that a single cycle of bortezomib does not seem to exert an effect on any long-lived antibody levels (further than 1 year post-transplant) whether the long-lived antibodies be DSA in sensitized kidney transplant recipients, anti-viral antibodies, or total immunoglobulins [20]. Yet, the group postulated that bortezomib may be more effective in more short-lived, intensely producing plasma cells as previously noted in the literature. Finally, as this is the only study to use bortezomib as solo immunotherapy, steroids may be critical for a synergistic, proapoptotic effect in normal plasma cells. 

## 5. Conclusions

Bortezomib's ability to target antibody producing plasma cell has generated interest in its use for pre-transplant desensitization and treatment of AMR. This drug may provide a promising insight into the management of patients undergoing kidney transplantation especially considering the large numbers of highly sensitized patients on the kidney transplant waiting list. New therapeutic strategies targeting reduction in DSA as well as managing AMR can provide opportunities for these patients. Although limited experience with bortezomib may seem to show promise in the realm of transplant recipients desensitization and treatment of AMR, there is also experience that may suggest otherwise. Specifically, the use of bortezomib with other accepted modalities for desensitization (PP, IVIG, and rituximab) may make it difficult to tease out bortezomib's role in transplant desensitization and treatment of AMR as highlighted in the limited reported instances of its use. Bortezomib's role in transplant desensitization may be better elucidated as more clinical data and well-designed clinical trials become available.

## Figures and Tables

**Table 1:** Clinical characteristics and outcomes of published desensitization/rejection protocols using Bortezomib as combination or solo therapy.

Author (reference)	Center	N	Indication^1^	Complete therapy	Results Summary	Conclusions
Wahrmann et al. 2010 [18]	Vienna, Austria	2	DS	(i) 2 cycles bortezomib at intervals of 3- and 4-months, both given with steroids	(i) cPRA mildly decreased in both patients (ii) Overall, no significant effect on the levels of antigen-specific IgG or ABO blood group antibodies	−

Walsh et al. 2010 [19]	Cincinnati Ohio, USA	2	AMR	(i) 1 cycle bortezomib (ii) ongoing plasmapheresis, rituximab, intravenous steroids (iii) pheresis done at least 72 hours post-bortezomib	(i) Immediate significant reduction of DSA (ii) Good allograft function at 5- and 6-months follow-up (iii) One patient had re-elevation of DSA which responded to a second course of treatment	+

Sberro-Soussan et al. 2010 [20]	Paris, France	4	AMR^4^	(i) 1 cycle bortezomib (solo therapy)	(i) No effect on anti-HLA antibodies within 40 subsequent days, and at 150 days follow-up.	−

Raghavan et al. 2009 [31]	Houston, TX, USA	1	DS	(i) 4 cycles bortezomib, one dose rituximab, daily mycophenolate	(i) Reduced PRA (55% → 30%) and significant reduction of class I antibodies (ii) Successful transplant with good allograft function at 6-months	+

Everly 2009 [14]	Cincinnati Ohio, USA	5	AMR	(i) 1 cycle bortezomib post treatment with “other” antihumoral therapies	(i) Median follow-up of 6.9 months. All patients had 50% reduction of DSA in 2-4 weeks. (ii) side effects in 4 of 5 patients (gastrointestinal, hematologic)^3^	+

Trivedi et al. 2009 [15]	Ahmedabad, Gujarat, India	11	Elevated DSA with MFI > 1000	(i) 1 cycle bortezomib followed by 2-4 treatments plasmapheresis and steroids (ii) 6 of 11 patients received one dose rituximab	(i) Follow-up at least 80 days post treatment. All patients had reduction in MFI within 4 weeks. (ii) 7 of 11 patients had reappearance of anti-HLA antibodies	+/−

Perry et al. 2009 [16]	Rochester, MN, USA	2	AMR	(i) 1 cycle bortezomib, daily plasmapheresis and IVIG	(i) Resolution of Rejection Peak MFI 13k and 14k^2^ At 1-year follow-up: MFI zero at and serum creatinine 0.6 and 1.3 mg/dl	+

Idica et al. 2008 [17]	??	13	DS	Details not apparent from article	(i) 10 of 13 had significant decrease (reversal) of DSA (ii) 100% had reduced MFI of antibodies	+

^1^DS: Desensitization, AMR = Antibody Mediated Rejection, DSA: Donor Specific Antibodies (elevated antibodies, but no clinical rejection). ^2^MFI: Mean Fluorescence Index. ^3^Side effects mentioned include thrombocytopenia and gastrointestinal toxicities. ^4^Patients had subclinical antibody mediated injuries with persistent DSA; hence it was acceptable to use bortezomib as solo therapy.
